# Temporal profiling of depression vulnerability in a preclinical model of sustained depression

**DOI:** 10.1038/s41598-017-06984-5

**Published:** 2017-08-17

**Authors:** D. Riga, L. J. M. Schmitz, W. J. G. Hoogendijk, A. B. Smit, S. Spijker

**Affiliations:** 1 Department of Molecular and Cellular Neurobiology, Center for Neurogenomics and Cognitive Research, Amsterdam Neuroscience, VU University, Amsterdam, The Netherlands; 2 Department of Psychiatry, Erasmus University Medical Center, Rotterdam, The Netherlands

## Abstract

Major Depression is a prevalent mental disorder that is characterized by negative mood and reduced motivation, and frequently results in social withdrawal and memory-related deficits. Repeated stressors, such as adverse life events, increase the risk for development of the disorder. Consequently, individual variability in stress response greatly weighs on depression-vulnerability and -resilience. Here, we employed the social defeat-induced persistent stress (SDPS) paradigm to identify depression-prone individuals and to examine the temporal development of depression in the months following exposure to brief defeat stress. Male Wistar rats were socially defeated (5 defeat episodes) and single-housed for a prolonged period of time (~24 weeks). We assessed the emergence of a sustained depressive-like state by repeatedly evaluating social motivation (social approach avoidance) and spatial memory (object place recognition) in SDPS rats during the isolation period. Individual variability in the effects of SDPS yielded two extreme subpopulations: an SDPS-prone group that showed gradual affective and cognitive deterioration in terms of social approach and memory retention, and a SDPS-resilient group that did not develop this phenotype. Notably, in SDPS-prone individuals, the affective deficits preceded later cognitive impairments, providing a novel temporal profile of the development of pathology in this preclinical model of sustained depression.

## Introduction

Major Depressive Disorder (MDD) is considered one of the most debilitating psychiatric disorders, ascribed to a ~16% lifetime prevalence and ~60% probability for severe clinical manifestation^1^. According to the Diagnostic and Statistical Manual of mental disorders (DSM-5), MDD is characterized by a variety of psychological, somatic, and social deficits^2^. Amongst them, decreased mood and diminished interest for pleasurable activities (anhedonia) are at the core of the depressive state^2^, frequently accompanied by impaired cognitive function^3^.

Social withdrawal, defined as disengagement from social activities that leads to impoverished interpersonal relationships, is a common symptom in depression^2^. Depressed patients often display diminished motivation for social interaction, which augments their subjective feelings of loneliness, in turn intensifying their depressed mood^4^. It is thought that social withdrawal results from the lack of reinforcement normally achieved by maintaining healthy interpersonal relationships, reflecting anhedonia^5, 6^. In addition, social withdrawal might emerge as a result of affiliation problems^7, 8^, when depressed individuals experience negative feelings, such as social anxiety, inferiority and detachment, upon exposure to social settings. Integrating these two hypotheses, it is proposed that deficits in approach-avoidance behaviour eventually culminate in social withdrawal^9^. In agreement, depression severity is predictive of diminished approach towards stimuli of positive valence^10^, resulting in greater withdrawal magnitude.

Cognitive deficits in depression include alterations in executive function, attention and memory that significantly interfere with a patient’s daily activities^11^. Memory deficits, such as difficulty in recollection and declarative memory, heavily depend on aberrant function of the hippocampus^12, 13^, in which depression-induced structural and functional alterations are well-described^14, 15^. In support, depressed patients display impaired spatial memory performance in virtual reality navigation tasks^16, 17^, which is accompanied by functional deterioration of the hippocampus^17^. Currently, it is unclear whether this cognitive dysfunction renders individuals prone to depression or whether cognitive deficits appear following the first depressive episode^18, 19^. It is suggested that cognitive impairment is a core feature of depression that develops independently of depressed mood, as it lingers in patients remitting from mood-related depressive symptoms^20, 21^. On the other hand, affective vulnerability, i.e., inability to regulate the emotional response, is shown to disturb cognitive function in depressed patients^22^, indicating a level of interdependency between the two symptoms.

Exposure to repeated stress is considered a primary trigger of the depressive state^23, 24^ and factors that regulate the stress response, ranging from genetic predispositions to social influences, have been implicated in vulnerability (or resilience) to depression^25^. It was postulated that abnormal reactivity to stress, such as excessive and/or prolonged hypothalamic-pituitary-adrenal (HPA) axis activation, increases the probability of depression onset and its magnitude^26^. In contrast, the employment of resilience-inducing strategies, e.g., adaptive HPA axis habituation, is thought to protect an individual from depression in case of severe or persistent stress^27^. These coping mechanisms are strongly determined by individual predispositions, such as trait anxiety, and their development is intertwined with the frequency of stress experience^28, 29^. During adverse life-events, proactive coping is associated with resilience and adaptability, whereas passive coping is thought to contribute to the development of stress-induced depression^30^. Supporting this notion, in individuals who adopt avoidance-related coping strategies, high frequency of negative life events predicts greater severity of depressive symptoms^31^.

Given its importance, individual variability in stress response and its association with the development of susceptibility or resilience to depression have been extensively studied at the preclinical level^27, 32^. This allowed for detailed examination of sub-phenotypes of the disease and for elucidation of depression comorbidities^33–35^. Notably, rodent models employing acute social defeat stress have successfully outlined brain pathways^36, 37^ and molecular mechanisms^38–40^ underlying stress vulnerability. We recently adopted a rat paradigm that combines acute social defeat stress with prolonged social isolation, the Social Defeat-induced Persistent Stress (SDPS) model^41^. SDPS induces a sustained depressive-like state that persists long after (>2 months) exposure to social defeat stress and emulates behavioural and physiological hallmarks of the human disease, such as anhedonia^42^, social withdrawal^43^, cognitive dysfunction^43, 44^ and hippocampal pathology^41, 45^.

Here, we investigated whether the SDPS paradigm can be used to identify depression-prone and -resilient individuals, and thus, to facilitate the characterization of depressive-like symptoms that develop over time. Depression susceptibility was estimated based on approach-avoidance behaviour and short-term spatial memory retention, in terms of social withdrawal (affective function) and depression-induced memory deficits (cognitive function), respectively. Furthermore, we examined the temporal profile of these SDPS-triggered impairments acutely after short but severe stress exposure (social defeat) and in the following months, in the presence of a constant subthreshold stressor (social isolation).

## Results

### Individual variability in the effects of SDPS

**Figure 1:**
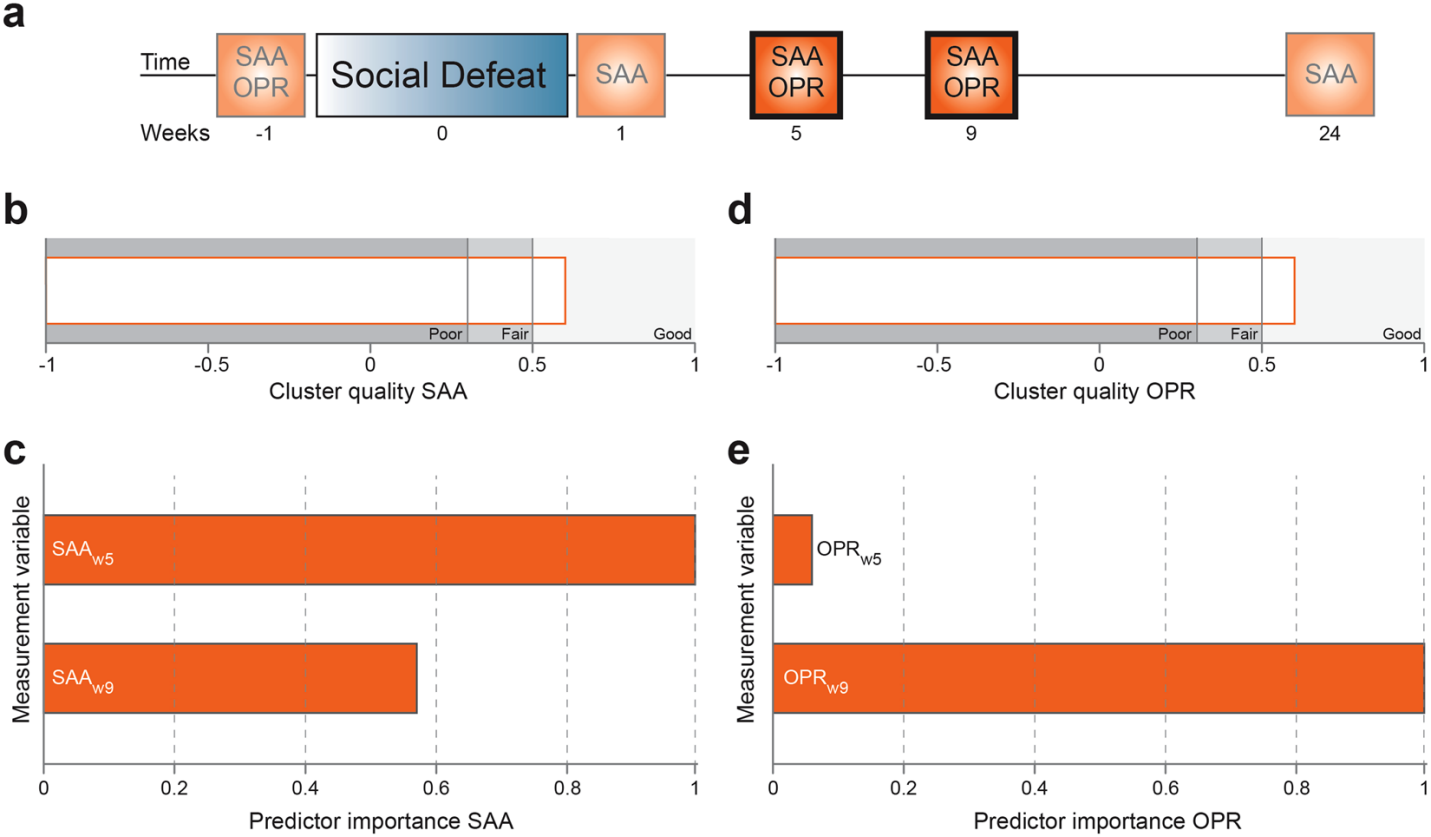
Cluster analysis of depressive-like behaviour over time: Overall model fit and predictor importance. (**a**) Experimental time-line of temporal profiling of affective and cognitive behaviour. Social approach-avoidance (SAA) and object place recognition (OPR) tasks took place in the weeks (w-1, w1, w5, w9, w24) before/after 5 daily defeat sessions (week 0). SDPS-vulnerability was estimated by a two-step cluster analysis of SAA and OPR performance as assessed at weeks 5 and 9 post-defeat (highlighted), using the Schwarz’s Bayesian criterion^35^. (**b**) Cluster quality for affective function (SAA) showed a good overall model fitting (0.60, orange open bar) when including individual data (n = 48) from the SAA_w5_ and SAA_w9_ tests. For comparison, poor (0.30) and fair (0.50) model fitting are depicted (grey shading). (**c**) From the two time points, performance at SAA_w5_ was the most important predictor of SDPS-proneness (predictor importance SAA_w5_, 1.00 *vs*. SAA_w9_, 0.57), indicating that SDPS-induced affective deficits that distinguish SDPS-prone from SDPS-resilient animals emerge at 1 month following the last defeat exposure. (**d**) Cluster quality for cognitive function (OPR) showed a good overall model fitting (0.60, orange open bar) when using individual data (n = 48) from the OPR_w5_ and OPR_w9_ tests. (**e**) From the two time points, performance at OPR_w9_ was the most important predictor of SDPS-proneness (predictor importance OPR_w9_, 1.00 *vs*. OPR_w5_, 0.06), indicating that cognitive deficits that distinguish SDPS-prone from SDPS-resilient animals develop later, namely at 2 months following the last defeat exposure.

As both affective and cognitive deficits determine the development and persistence of the depressive state^21, 46^, we used the performance of SDPS animals in the social approach avoidance (SAA) and object place recognition (OPR) tasks at 2 different time points (week 5 (w5) and 9 (w9) after defeat) to identify subpopulations of SDPS-prone and SDPS-resilient individuals in a large cohort of animals^35, 39^ (Fig. 1a). Based on this, we used data clustering for identification of two clearly divergent groups, in terms of affective and cognitive performance, as described below in detail. Individual data for both tests and all time points are presented in Supplementary Figure S1.

#### SDPS effect on approach-avoidance behaviour

**Figure 2:**
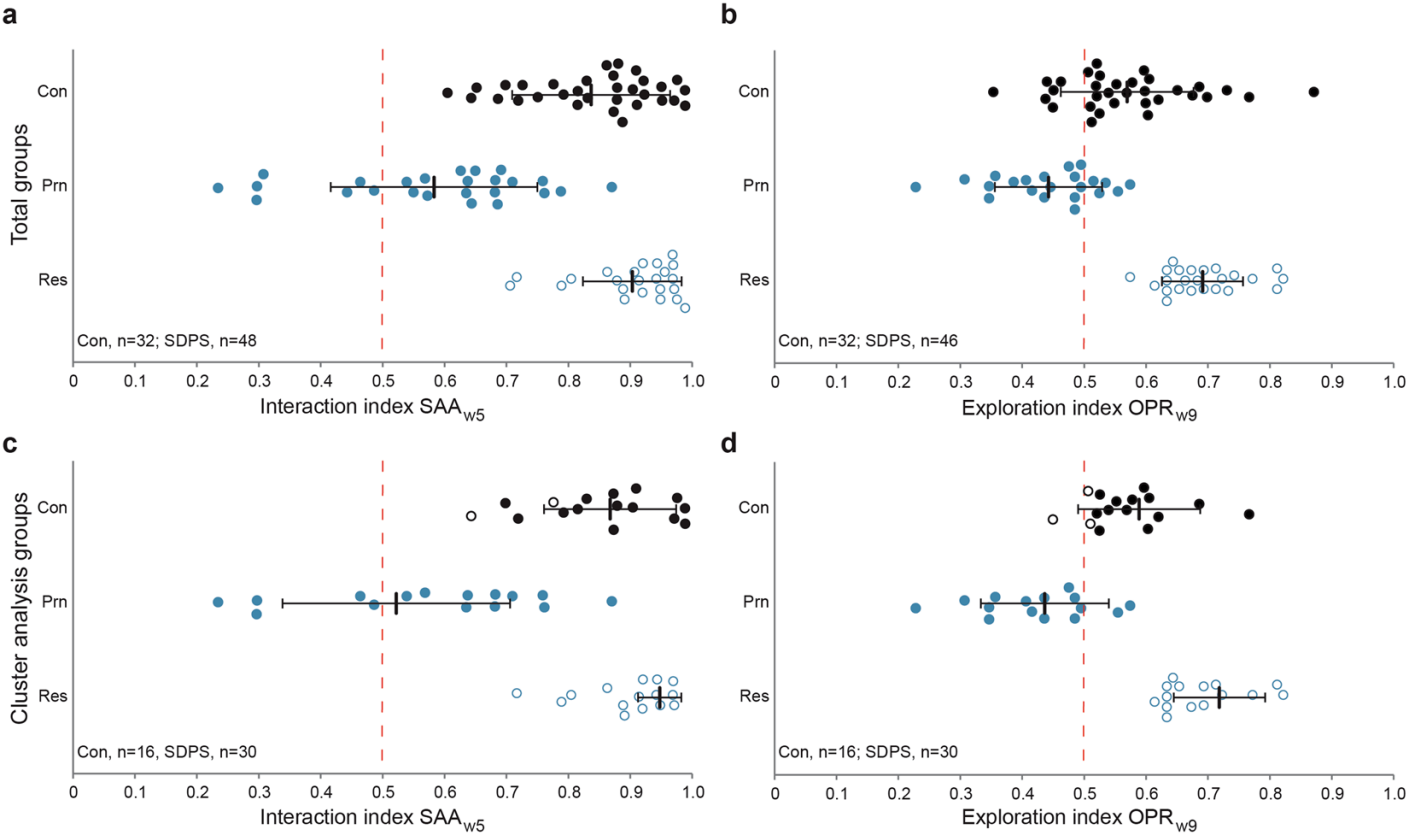
Cluster analysis: Individual variability in the effects of SDPS on the affective and cognitive domain. Two-step cluster analyses of social approach avoidance (SAA) and object place recognition (OPR) performance, each at weeks 5 and 9 post-defeat, revealed the emergence of two distinct SDPS groups, one displaying severely disrupted behaviour (SDPS-prone; Prn) and one exhibiting performance similar to controls (SDPS-resilient; Res). Individual performance at the SAA_w5_ (**a**,**c**) and OPR_w9_ (**b**,**d**) tests is depicted, as cluster analyses indicated these particular tests as the most important predictors of SDPS-vulnerability (*cf*. Fig. 1). (**a**,**b**) Individual performance at SAA (**a**) and OPR performance (**b**) on the total set of SDPS rats (n = 46–48), and the total set of control (Con) rats (n = 32) for comparison. (**c,d**), Individual performance of control (Con) rats (n = 16) and SDPS rats showing overlap in SAA and OPR after cluster analysis (see (**a**,**b**); n = 30; n = 15 per subgroup) at the SAA (**c**) and OPR (**d**) tasks. SDPS-resilient rats clustered together with all but two control rats at the SAA task (**c**), and all but three control rats at the OPR task (**d**), which are indicated by open circles. All panels: Group mean and standard deviation (vertical line with horizontal whiskers); red dashed line indicates interaction (**a**,**c**) or exploration (**b**,**d**) indices at chance level (0.50).

Based on clustering (Schwarz’s Bayesian criterion^35^) of approach-avoidance behaviour at two time-points after defeat (average model silhouette 0.60; Fig. 1b), the SAA_w5_ test was the most prominent predictor of SDPS-induced deficits in social motivation (predictor importance 1.00 *vs*. 0.57 for SAA_w9_, Fig. 1c), indicating establishment of affective vulnerability a month following defeat. Rats were clustered in two groups: 23 SDPS rats were identified as SDPS_SAA_-resilient (interaction index group mean: SAA_w5_, 0.91 ± 0.02; SAA_w9_, 0.92 ± 0.01), whereas the remaining 25 rats clustered in the SDPS_SAA_-prone group (interaction index group mean: SAA_w5_, 0.59 ± 0.03; SAA_w9_, 0.71 ± 0.03) (Fig. 2a). These two clusters showed distinct performance at the individual time points (SAA_w5_, U = 562.00, *P* < 0.001; SAA_w9_, U = 528.00, *P* < 0.001). Notably, SAA performance acutely following defeat was found to decrease the overall model fitting (Supplementary Fig. S2), suggesting that although immediate post-defeat SAA performance reflects the effects of acute defeat stress^39^, its relevance to predict the development of a long-lasting sustained depressed state is nominal.

#### SDPS effect on short-term object place memory

Using task performance at two time points (average model silhouette 0.60; Fig. 1d), the OPR_w9_ test was the most prominent predictor of SDPS effects on cognitive function (predictor importance 1.00 *vs*. 0.06 for OPR_w5_, Fig. 1e), indicating the establishment of cognitive vulnerability at 2 months following defeat. Supporting this notion, in the two identified clusters a significant between-group difference was observed only at the OPR_w9_ test: OPR_w5_, F(1,44) = 2.29, *P* = 0.137; OPR_w9_, F(1,44) = 122.03, *P* < 0.001. In particular, 24 SDPS rats were identified as SDPS_OPR_-resilient (exploration index group mean: OPR_w5_, 0.61 ± 0.03; OPR_w9_, 0.70 ± 0.01), whereas the other 24 rats clustered in the SDPS_OPR_-prone group (exploration index group mean: OPR_w5_, 0.55 ± 0.03; OPR_w9_, 0.45 ± 0.02) (Fig. 2b).

#### Selection of SDPS-prone *vs*. -resilient subpopulation

Clustering based on the SAA and OPR tasks showed a substantial overlap, indicating that SDPS-induced depression proneness was reflected in deficits of *both* the affective and the cognitive domain. Particularly, 65% of the SDPS_SAA_-resilient group was part of the SDPS_OPR_-resilient group, and likewise, 64% of SDPS_SAA_-prone animals were clustered within the SDPS_OPR_-prone group. Rats with overlap in both domains were assigned to the SDPS-prone and SDPS-resilient populations, resulting in a total of 15 rats per subgroup (Fig. 2c,d). Re-analysis of cluster data, this time including the individual scores from controls (n = 16), validated the final population division, as SDPS-resilient animals clustered in general together with controls in both tasks (SAA, all but 2 controls; OPR, all but 3 controls) (Fig. 2c,d).

### Temporal profile of SDPS-induced affective and cognitive deficits

#### Development of deficits in approach-avoidance behaviour

**Figure 3:**
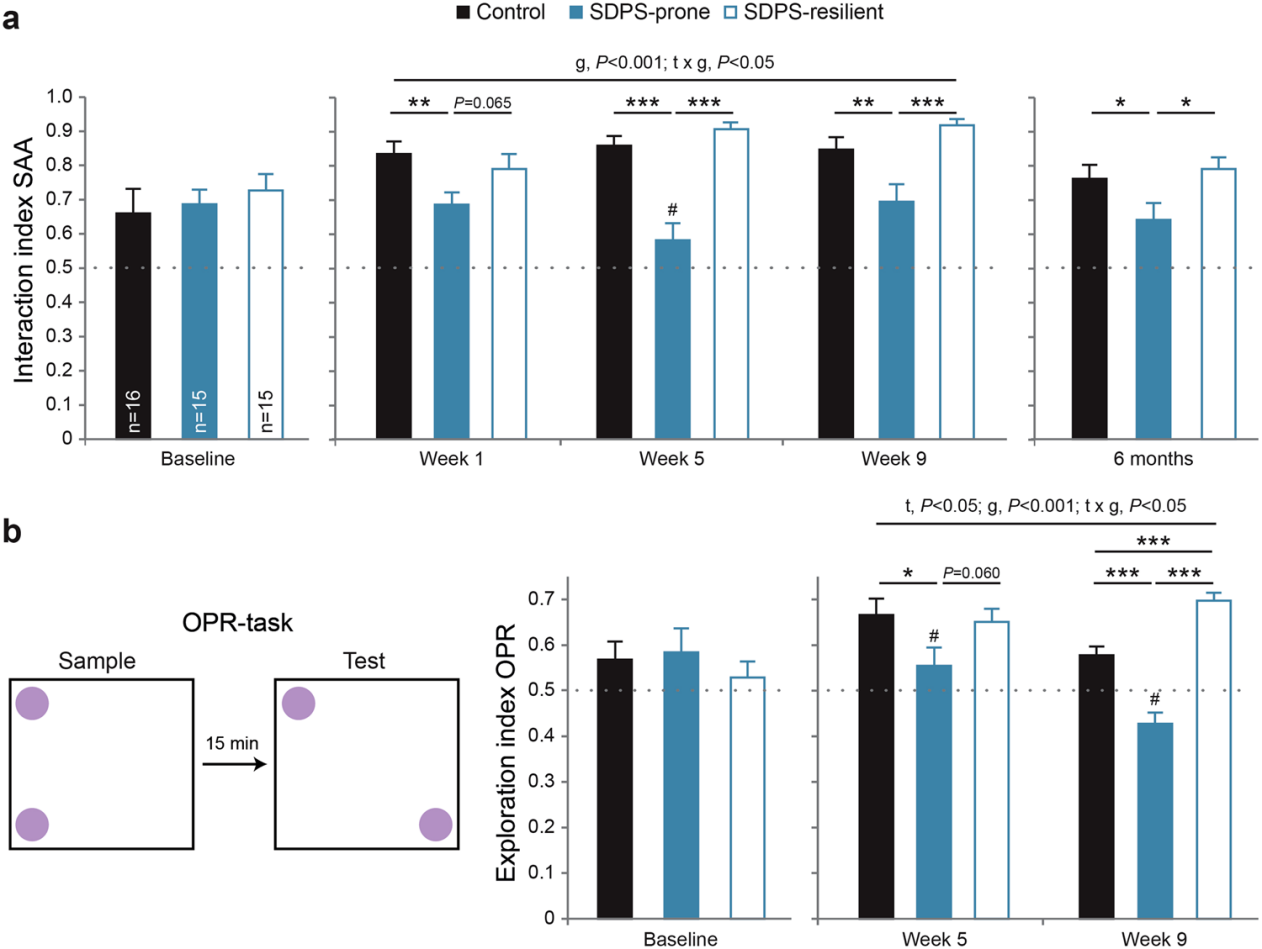
Development of affective and cognitive deficits in SDPS-prone and SDPS-resilient rats. (**a**) Approach-avoidance behaviour was examined in 5 subsequent SAA tests provided during a period of ~6 months (*cf*. Fig. 1a). Before defeat (baseline), no pre-existing differences were observed between groups. At SAA_w5_, only the SDPS-prone group failed to display preference for the social target. In the months following defeat, SDPS-prone rats showed reduced interaction index *vs*. controls and SDPS-resilient animals, indicating development of social withdrawal that persisted up to 6 months. (**b**) *Left*: Schematic representation of the object place recognition (OPR) task, with a 5-minute sampling phase, a 15-minute retention interval and the 5-minute testing phase, in which one of the two identical objects was displaced. *Right*: Short-term spatial memory was assessed in 3 OPR tests given during a period of 2 months (*cf*. Fig. 1a). Prior to defeat no between-group differences were seen. Already at 1 month from defeat, SDPS-prone animals showed inability to retain spatial information for a displaced object, which was exaggerated at the 2-month test. Both control and SDPS-resilient groups displayed intact memory retention. Repeated measures ANOVA main time (t), group (g) and time x group interaction (t x g) effects and pairwise comparisons are indicated. Dotted line represents chance levels (0.50) of interaction (SAA, **a**) or exploration (OPR, **b**). ^#^No preference for the social target (SAA) or the displaced object (OPR); *P < 0.050; **P < 0.010; ***P < 0.001.

In order to clarify the temporal profile of depression-associated deficits in social behaviour, SAA performance of SDPS-prone, SDPS-resilient and control groups was plotted in a retrospective manner (Fig. 3). This revealed that before the start of the SDPS paradigm, no between-group differences in baseline approach behaviour were observed between the three groups (F_SAA-bl_(2,43) = 0.36, *P* = 0.700; Fig. 3a). Following social defeat, SAA performance (repeated measures ANOVA: SAA_w1_, SAA_w5_, SAA_w9_) showed no effect of time (F_SAA_(2,86) = 1.61, *P* = 0.207). A significant group by time interaction and group effect were observed (F_SAAxGROUP_(4,86) = 2.75, *P* = 0.033; F_GROUP_(2,43) = 28.06, *P* < 0.001), indicating that SDPS differentially affected SAA performance in each group during the weeks after defeat (Fig. 3a).

In particular, already acutely after social defeat (F_SAAw1_(2,43) = 3.87, *P* = 0.029), SDPS-prone animals showed reduced approach behaviour when compared with controls (*P* = 0.009). A trend for differential performance between SDPS-prone and SDPS-resilient rats (*P* = 0.065) was observed, as the latter group performed similar to controls (*P* = 0.430).

Analysis of SAA_w5_ test (H = 24.45, *P* < 0.001) confirmed the establishment of social avoidance in SDPS-prone rats (*P* < 0.001 *vs*. control and SDPS-resilient). The resilient group escaped the effects of SDPS and performed similar to controls (*P* = 0.308). Comparable results were obtained when analysing SAA_w9_ data (H = 14.68, *P* = 0.001), with SDPS-prone animals showing reduced approach behaviour as compared with both controls (*P* = 0.005) and the SDPS-resilient group (*P* < 0.001). No difference in SAA performance between the latter groups was observed (*P* = 0.185). A positive correlation between w5 and w9 SAA tests further validated the stability of performance in SDPS rats over-time, namely, a sustained avoidance response in SDPS-prone rats *vs*. intact social approach in the SDPS-resilient group (Supplementary Fig. S3).

At ~6 months from the last defeat exposure, a significant group effect (F(2,43) = 3.53, *P* = 0.038) confirmed that SDPS-prone animals continued displaying reduced social approach as compared with controls and SDPS-resilient rats (*P* = 0.046 and *P* = 0.017, respectively, Fig. 3a). Control and SDPS-resilient animals exhibited similar approach-avoidance performance (*P* = 0.634).

#### Development of deficits in short-term object place memory

Similar to SAA, the performance of the SDPS-prone, SDPS-resilient and control groups at the OPR task was used to illustrate the temporal profile of depression-associated cognitive deficits. No between group effects were observed in short-term memory retention before the start of the SDPS paradigm (F_OPR-bl_(2,41) = 0.43, *P* = 0.651, Fig. 3b). Following social defeat, overall OPR performance (repeated measures ANOVA: OPR_w5_, OPR_w9_) showed significant effects of time (F_OPR_(1,42) = 5.83, *P* = 0.020), group (F_GROUP_(2,42) = 20.95, *P* < 0.001) and interaction (F_OPRxGROUP_(2,42) = 5.06, *P* = 0.011). This indicated differential OPR performance of each group over the course of the two months after defeat (Fig. 3b).

In particular, at one month following defeat, a trend for a group effect (F_OPRw5_(1,42) = 2.92, *P* = 0.065) was observed, which was driven from the considerably poorer OPR scores of SDPS-prone rats compared with the other two groups (*P* = 0.031 *vs*. control; and *P* = 0.060 *vs*. SDPS-resilient). As with the previous tests, SDPS-resilient animals did not differ from controls (*P* = 0.769). Likewise, a significant group effect was observed at the OPR_w9_ test (F_OPRw9_(1,43) = 42.76, *P* < 0.001), with SDPS-prone rats displaying a significantly lower exploration index compared with both control and SDPS-resilient groups (*P* < 0.001 *vs*. both). This confirmed the consolidation of cognitive deficits in the SDPS-prone group at two months following exposure to defeat stress, which coincided with impaired social behaviour (Supplementary Fig. S4). Surprisingly, a significant group effect was observed between control and SDPS-resilient rats (*P* < 0.001), reflecting a slight improvement of OPR performance in the SDPS-resilient group (paired t-test OPR_w5,w9_, t(14) = −1.58, *P* = 0.136) together with a decrease in performance of controls (paired t-test OPR_w5,w9_, t(14) = 2.58, *P* = 0.022) (Fig. 3b).

Together, the SAA and OPR data indicated the formation of two distinct subpopulations following social defeat, which was independent of baseline performance. The SDPS-resilient population coped with defeat and isolation stress and did not develop any of the affective or cognitive deficits commonly seen after SDPS^42, 43^. In contrast, the SDPS-prone population showed long-lasting deterioration of affective performance, reflected in social withdrawal, and was accompanied by severe impairments in spatial memory, which worsened over time. Individual-based analysis of the temporal progression of the depressive-like state argued in favour of early establishment of impairments in social behaviour and later coincidence of the affective-cognitive symptoms.

#### Submission latency

**Figure 4:**
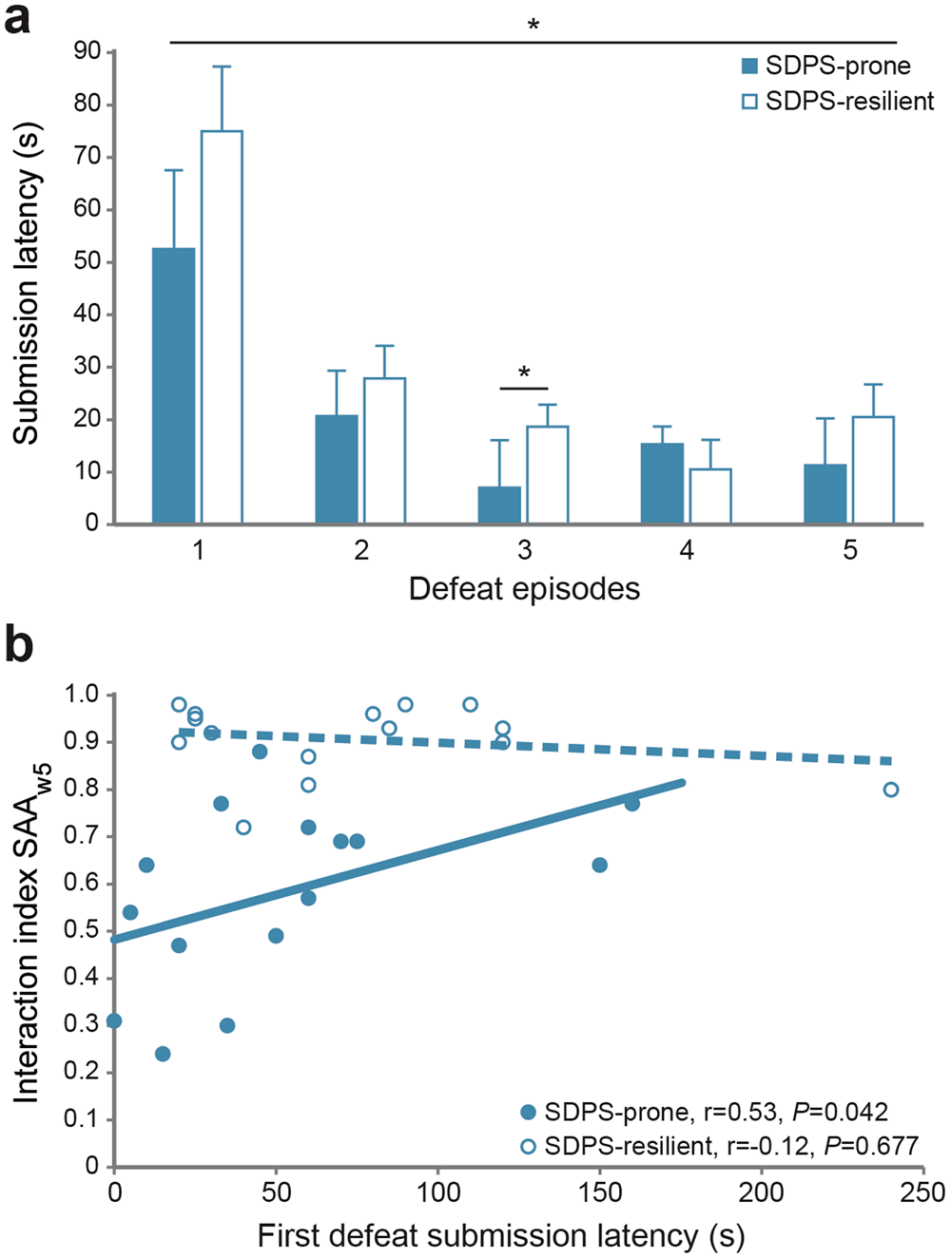
Submission latency during social defeat. Latency to assume the first submissive posture during the five defeat sessions was documented for each SDPS animal. (**a**) Following the two-steps cluster analysis, average latency to first submission was calculated for each of the emerging SDPS-prone and SDPS-resilient subgroups. SDPS-prone animals displayed shorter submission latency over the five defeat sessions and this effect was most pronounced in day 3 of the social defeat period. (**b**) In the SDPS-prone group, submission latency during the first defeat episode was positively correlated with social avoidance as assessed at the SAA_w5_ test, which was the most prominent predictor of SDPS-proneness in the affective domain. No such correlation was observed for the SDPS-resilient rats. Spearman’s rank correlation coefficient (r) and the correspondent *P*-value is indicated for each group. *P < 0.05.

Coping strategies during exposure to defeat stress, e.g., counter-attacks or freezing, predict the duration and severity of the psychobiological effects of defeat^47^. Therefore, we examined latency for first submission during defeat in the two subpopulations identified as SDPS-prone and SDPS-resilient. Analysis of submission latency over the five defeat episodes revealed a significant between-group effect (Friedman’s χ^2^(4) = 45.89, *P* < 0.001), as SDPS-prone rats submitted faster *vs*. their SDPS-resilient counterparts (Fig. 4a). Notably, a positive correlation between submission latency during the first defeat session and performance at SAA_w5_ was observed that was specific for the SDPS-prone group: SDPS-prone, Spearman’s r(15) = 0.53, *P* = 0.042; and SDPS-resilient, Spearman’s r(15) = −0.12, *P* = 0.677 (Fig. 4b). No correlation between submission latencies and any of the other behavioural tests (SAA_w1_, SAA_w9_, OPR_w5_, OPR_w9_) was seen.

## Discussion

In the present study, a large group of rats (n = 48) was subjected to the SDPS paradigm, with 5 daily defeat sessions followed by prolonged single-housing (6 months). The depressive-like state, which is known to be long-lasting^41–43^, was assessed at different time points after social defeat while animals remained in isolation. This entailed repeated measuring of social approach-avoidance behaviour (SAA) and performance in a short-term spatial memory task (OPR) with sufficient inter-test time interval. By adopting this approach, we examined how affective deficits, in the form of reduced motivation for social interaction, and cognitive deficits, seen as failure in memory retention, develop over time with a focus on the individual.

Individual variability in the effects of SDPS on each parameter was assessed by cluster analysis, revealing two distinct subpopulations of SDPS-prone and SDPS-resilient rats. This was in absence of pre-existing behavioural differences, suggesting that reactivity to social stress determines later depression vulnerability. SDPS-proneness was associated with persistent social withdrawal and a progressive decline in spatial memory. Furthermore, temporal profiling of SDPS effects showed that affective deficits emerged first, whereas aberrant memory processes developed later. SDPS-resilience was associated with absence of depression-like deficits.

### Individual variability in the effects of SDPS on affective behaviour

Reduced interest in social activities is one of the core symptoms of depressive pathologies^2^, whereas loneliness and perceived isolation from social contexts contribute to chronic depression in humans^48, 49^. Reduced social motivation and increased social anxiety are risk factors for the onset and the duration of depression^50, 51^. Likewise, avoidance response, which precedes social withdrawal, confers vulnerability to the development and persistence of the disorder^9^. As such, social withdrawal, in the form of reduced interaction with an unfamiliar social target, has been extensively used to assess the development and magnitude of depressive-like states at the preclinical level^52^.

We previously reported that a general population of SDPS rats exhibits persistent social withdrawal that lasts up to 6 months^43^. This reliably resembles social withdrawal during development, in which severe reticence is longitudinally present^53^. Here we confirmed the negative effects of SDPS on affective behaviour, showing its importance in the development of depression vulnerability. SDPS-prone rats showed decreased approach behaviour immediately after the defeat week (SAA_w1_) and displayed reduced social motivation up to 6 months following the last defeat exposure (SAA_6mth_). The acute effect of social stress (SAA_w1_) in the SDPS-resilient rats was mild, as their interaction scores reached midway that of the control and SDPS-prone groups. Thereafter, the SDPS-resilient population showed stable approach behaviour throughout the experimental design, similar to controls. Together, these data suggest a disrupted affective response in SDPS-prone rats that leads to permanent social deficits, an effect absent in SDPS-resilient animals.

### Individual variability in the effects of SDPS on cognition

In recent years, impaired cognition in MDD, including attentional bias and poor working memory^11, 54^, has gained growing attention in the clinic, as it is thought to perpetuate the depressive state and to hamper recovery^3, 55^. Longitudinal studies support a unidirectional relation between depression and cognitive dysfunction, with pre-existing depressive symptoms accelerating global cognitive deficits and episodic memory problems^18^. In addition, depression recurrence predicts failure in recollection memory^56^ and a parallel reduction in hippocampal volume^15, 57^, indicating that the duration and persistence of the depressive state negatively impact on brain morphology and cognitive function.

We previously showed that SDPS induces prolonged cognitive dysfunction, reflected in deficits in hippocampus-mediated spatial memory^42, 43^. Here, we validated the detrimental impact of depression-triggering stressors on hippocampal function^58^. Similar to approach-avoidance behaviour, only the subpopulation of SDPS-prone animals showed this inability to retain short-term information with regard to the spatial location of an object. SDPS-resilient animals were protected from SDPS-induced memory deficits, showing a relative improvement in OPR performance at two months following defeat.

The most prominent reduction in spatial memory performance was observed in the SDPS-prone group at two months after defeat, mimicking progressive cognitive decline in presence of a sustained depressive-state. Cognitive deficits following acute social defeat stress include reduced memory performance at the novel object recognition task^59^, which is largely independent of hippocampal function^60, 61^. In contrast, performance at the Morris water maze task, which, similar to OPR, examines hippocampus-mediated spatial memory, is not affected in the first two weeks following exposure to defeat stress^59^. In addition, in rats exposed to social defeat stress, long-term spatial memory deficits, as examined in the radial arm water maze, appear following >1 month from the last defeat exposure^62^.

In the present study, we did not assess OPR performance acutely following a defeat episode. Thus, based on our data we cannot exclude effects of acute stress on short-term recognition memory. In fact, it is very likely that spatial memory is affected at these early time-points after stress, as reviewed for several other stressful paradigms in rodents^63^. It is worth noting that these acute stress effects are mediated via glucocorticoid signaling^64^, which is altered shortly following exposure to stressful stimuli, including social defeat stress^65, 66^. However, as we did not observe any difference in basal glucocorticoid levels long-term after defeat^41^, we consider that the progressively deteriorating spatial memory performance we report here is mediated via divergent mechanisms, which apparently develop over time in absence of stress.

Taken together, it is plausible that maintained disruption of hippocampus-mediated cognition necessitates the presence of a chronic depressive-like state, just as observed in humans. Furthermore, our data indicate that, similar to vulnerability, resilience to depression-induced cognitive disturbances is an active process relying on adaptations that evolve over lengthy periods of time.

### Temporal profiling of depressive-like symptoms in SDPS-prone individuals

Currently, profiling of depressive symptoms is limited to the prerequisite of experiencing severe mood- *or* anhedonia-associated impairments that persist for more than 2 weeks^2^. This neglects temporal aspects of depression occurrence and disease trajectory^67^. Likewise, most preclinical research relies on acute one-off behavioural assessments of the depressive-like state, seemingly overlooking empirical data that suggest that depressive pathology and the related burden intensify with time, including frequency of depressive episodes and their duration^68^.

Here, by employing repeated measurements of depression-associated deficits in two distinct behavioural domains, we provide evidence for a unique temporal profile in the development of the depressive-like state in SDPS-prone individuals. As the predictor efficacy of the cluster analyses revealed (*cf*. Fig. 1), there is a clear distinction in the development of the affective and cognitive phenotypes over time. Deficits in affective behaviour, i.e., reduced interest for social interaction (SAA test) appeared first, and were able to distinguish depression-prone animals already at week 5 following exposure to defeat stress. Cognitive impairments, i.e., reduced short-term spatial memory retention, developed later, as depicted by the strong influence of OPR_w9_ in predictor efficacy. This temporal profile was verified by a significant positive correlation between early affective and late cognitive deficits (Supplementary Fig. S5), indicating that the magnitude of impairments in social behaviour could predict the severity of cognitive symptoms in depressed individuals. From a clinical perspective, our results argue in favour of early identification of patients with mood-related symptoms and their recruitment for specific programs, such as prevention of social isolation and stimulation of cognitive capacity.

In inbred mice that are identified as susceptible based on increased avoidance behaviour at the SAA task shortly after social defeat, exposure to social stress halts normal hippocampal growth compared with the resilient subpopulation^69^. In addition, susceptible mice show pre-existing hippocampal volume differences that correlate with post-stress avoidance performance^69^. Together these data suggest that epigenetic, stress-induced hippocampal susceptibility can confer depression vulnerability. Although cognitive function was not assessed in these animals, these results fit in the temporal profile of the depressive state illustrated in our study, with the effects of SDPS first manifested in social avoidance and later, possibly following structural and functional reorganization of the hippocampus, in cognitive decline. This is in accordance with the idea that affective disturbances precede, or might even promote, deficits in cognitive processes in depression^22, 70^.

### Individual differences in coping styles during social defeat

Coping strategies highly influence one’s ability to adapt during exposure to severe stress, and trigger allostatic mechanisms serving resilience or promoting vulnerability^27^. At the preclinical level, during social defeat, active (confrontation, defensiveness) or passive (immobility, submission) coping styles have been reported^71–73^ and are considered to be closely associated with responsivity to social defeat stress^74^ and to subsequent stressors^75^. A well-established measure of coping style during defeat stress is the latency to assume a subordinate posture^47, 76^, which has been used before in order to distinguish defeat-prone from defeat-resilient individuals^30^. In the present study, SDPS rats that were identified as prone following cluster analysis showed faster submission latency *vs*. their resilient counterparts. Our data are in agreement with the notion that rodents that exhibit passive coping styles, such as quick subordination during defeat, display vulnerability to depression, just like humans^31^.

Behavioural readouts that promote initiative and free choice are most discriminative of a proactive *vs*. a reactive coping style in face of stress^32^, granting the SAA task with high face value in categorizing active *vs*. passive copers. Following SDPS, latency to first submission predicted avoidance performance at the five weeks SAA task. Notably, this positive correlation was selective to the SDPS-prone subpopulation, further supporting an interplay between passive coping strategies and later vulnerability to the depression-triggering effects of stress^31, 71^.

## Conclusions

In order to further elucidate the underlying causes of depression and to provide successful therapeutic options to treatment-resistant individuals^77–80^ preclinical models should prioritize on individual variability to the lasting effects of stress. Our data argue for the need of a temporal analysis of both affective and cognitive disturbances in paradigms that model (vulnerability to) depression. Finally, our data suggest that affective/motivational deficits precede cognitive decline in depression, which could prove useful in designing preventive and treatment strategies against this debilitating disorder.

## Animals, Methods and Materials

### Animals and social defeat-induced persistent stress (SDPS)

SDPS was carried out with male Wistar rats (n = 48 defeat, n = 32 controls, 9–10 weeks of age) as described before^42, 43^ (Supplemental Methods). In brief, SDPS rats were exposed to five 15-minute daily social defeat sessions as follows: rats were transported to the residents’ housing room and placed inside the residents’ cages (defeat cage). A transparent, perforated plexi-glass partition wall was used to separate the residents from the intruders, allowing for sensory exchange, but not for physical contact (pre-fight phase, 5 minutes). The wall was removed and Wistar rats were then exposed to a 5-minute fight phase, during which they were forced into submission. The defeat session concluded with an additional 5-minute period, during which the partition wall was placed back, separating the resident from the intruder (post-fight phase). A different resident was matched to each Wistar rat per day. From the first defeat session onwards, all animals were single-housed and remained in social isolation for the rest of the experimental manipulations, in absence of further sensory interaction with the stressor (residents), in a separate housing room. Two researchers monitored the social defeat sessions and the latency to submission during the fight phase was recorded for each rat in each of the five sessions provided. Experiments were divided over 3 independent batches, separated by 1 week each. Animals were housed on a reversed 12-h light-dark cycle (lights on 19.00 h) and all experiments were conducted during the dark phase. Rooms were equipped with infrared lights, which Wistar rats cannot detect. Animals received food and water *ad libitum*. All experiments were approved by the VU University Amsterdam Animal Users Care Committee, and were performed in accordance with the relevant guidelines and regulations.

### Assessment of the depressive-like state

#### Social approach-avoidance test (SAA)

Approach-avoidance behaviour was estimated using an unfamiliar Long-Evans adult male rat (resident) as previously described^42, 43^ (Supplemental Methods). Interaction index was calculated as time spent in active zone (resident zone)/total exploration time (resident + neutral zone), in a 5-minute test. In order to examine the development and progression of social withdrawal the weeks after social defeat, all animals were exposed to 5 consecutive SAA tests: the week before social defeat (baseline, bl); following the defeat week (acute, w1); at week 5 (w5); at week 9 (w9) and at 6 months (6mth) following the last defeat exposure.

#### Object place recognition (OPR)

Hippocampus-dependent short-term memory was assessed by the object place recognition task using a 15-minute retention interval as previously described^42, 43^. Discrimination between the spatial locations of the two objects was used to assess spatial memory (exploration index = time spent in novel location/total exploration time (novel + familiar location)) in a 4-minute test. In order to examine the development and progression of cognitive impairments after SDPS, all animals participated in three OPR tests given the week before social defeat (baseline, bl); at week 5 (w5) and at week 9 (w9) following the last defeat exposure.

### Statistical analyses

#### Analysis of behavioural readouts

All behavioural data collected from SAA, OPR were analysed using repeated measures analysis of variance (ANOVA), with test (time-points) as within- and group as between-subject factors. When *P*-values reached level of significance (*P* < 0.05), further analysis was performed using one-way ANOVA, paired or unpaired student’s t-test and post-hoc Tukey-HSD multiple comparisons. Homogeneity of variance, sphericity and normality assumptions were estimated and Huynh-Feldt correction or the non-parametric Kruskal-Wallis H, Mann-Whitney U and Friedman χ^2^ tests were implemented in case of violation. Preference in interaction and exploration indexes (SAA, OPR) was estimated against a fictive group representing performance at chance levels, while retaining the same variation as the experimental groups^81^. Correlation between different experimental readouts was estimated using Spearman’s correlation coefficient (r). All statistics were performed using IBM SPSS Statistics 21. All group data are depicted as mean ± SEM.

During assessment of the depressive-like state, the tracking software was erroneously terminated, leaving datasets for the following tests incomplete: ORR_bl_, n = 2; OPR_w5_, n = 3.

#### Selection procedure

SDPS rats were assigned to either SDPS-prone or SDPS-resilient subgroups following a two-step cluster analysis of individual performance in the social-approach avoidance test (SAA), and object place recognition test (OPR) at two time points, namely at week 5 (w5) and week 9 (w9) after the last defeat exposure. Both behavioural readouts (SAA, OPR) were weighted equally for final group assignment, as the criterion for susceptibility or resilience required to include both affective and cognitive aspects of the depressive-like state. Cluster analysis was performed using IBM SPSS Statistics 21, based on the Schwarz’s Bayesian criterion^35^ and with automatic generation of cluster numbers to avoid biased subject selection. First, we performed cluster analysis using the SAA data and SDPS animals were classified as prone or resilient based on their motivation to interact with the social target (affective domain). Subsequently, OPR data were used for cluster analysis in order to identify prone *vs*. resilient individuals in respect to spatial memory retention (cognitive domain). Animals that showed overlapping clustering in the two cluster analyses were finally identified as SDPS-prone (n = 15) and SDPS-resilient (n = 15).

Control animals were divided in two equally performing groups (balanced average performance in SAA and OPR tests^43^). Thus, a total of 16 control rats participated in the experiments described above, whereas the other 16 served as the control group in our previous study^43^. Data obtained from the final SDPS-prone and -resilient groups were re-analysed together with controls to validate the particular approach, i.e. post-hoc fitting of the clustering method (*cf*. Fig. 2).

### Data Availability

The datasets generated during and/or analysed during the current study are available from the corresponding author on reasonable request.

## Electronic supplementary material

Supplementary information
