# Supplementary material for: Temporal profiling of depression vulnerability in a preclinical model of sustained depression
Source: Sci Rep. 2017 Aug 17;7:8570. doi: 10.1038/s41598-017-06984-5 (PMC5561121; doi:10.1038/s41598-017-06984-5)
Supplement: Supplementary file 1 — Supplementary information [file 41598_2017_6984_MOESM1_ESM.pdf]

# Temporal profiling of depression vulnerability in a preclinical model of sustained depression

Riga D[1], Schmitz LJM[1], Hoogendijk WJG[2], Smit AB[1], Spijker S[1,*]

## Supplemental Methods & Material

<u>Animals and social defeat-induced persistent stress (SDPS)</u>

Pair-housed male Wistar rats (Harlan CPB, Horst, Netherlands) 6–7 weeks old, weighing <200 g upon arrival were habituated in the vivarium and to the reversed light-dark cycle (2 weeks) and subsequently exposed to social defeat-induced persistent stress (SDPS, ≥10 weeks old)[1,2]. Male Long-Evans rats, (Charles River, UK, weighing >500 g) that were paired-housed with age-matched tube-ligated females (Wistar, Harlan) were used as residents. Long-Evans were screened for territorial behaviour and dominant residents that refrained from inducing physical injuries to the intruders were used for social defeat. In defeat days, females were removed from the Long-Evans cages, at least 15 minutes before the start of the resident-intruder protocol. In defeat days, control animals (n=32) were transported to the residents' housing room and allowed to explore an empty defeat cage for 15 minutes. Due to the large number of animals included in the initial experiment, social defeat and subsequent behavioural assessment were performed in 3 consecutive weeks, as animals arrived in the vivarium over 3 independent batches, separated by 1 week each. No between-batch effect in behaviour was observed.

<u>Assessment of the depressive-like state</u>

Before participating in the behavioural assays, all animals were transferred to the video-recording room and habituated to the test arena (plastic, opaque, 79 x 57 x 42 cm) for at least 10 minutes during 3 consecutive days. Animals were subjected to the Social Approach-Avoidance (SAA) and the Object Place Recognition (OPR) tasks at different time points, as indicated in Figure 1a. All video recordings were analyzed with Viewer2 software (BiObserve GmbH, Bonn, Germany).

*Social approach-avoidance test (SAA)* – Rats were habituated and allowed to explore two empty target boxes (TBs, perforated, metal, 16 x 7 x 8 cm) located in the opposite sides of the testing arena (sample phase). Subsequently, an unfamiliar resident was introduced to one of the TBs and rats were allowed to explore and interact with the target, in absence of direct physical contact (test phase). Interaction zones were represented within 2 cm distance from each TB and duration of interaction was based on tracking of the nose of the animal (rat facing the TBs). Active and inactive zones were randomly assigned, in all tests provided and between groups, to avoid development of preference.

*Object place recognition (OPR)* – Following habituation, rats were allowed to explore two identical objects (cylinders or cubes, metal, 8 x 8 x 35 cm), located in two opposite corners of the arena (sampling phase). After a 15-minute time interval, both objects were replaced with 2 identical ones, and one was displaced to a different position. Exploration zones were represented within 2 cm distance from each object and duration of exploration was based on tracking of the nose of the animal (rat facing the objects). The position of novel and familiar locations and the choice of object shapes were random in all tests provided and between groups, to avoid development of preference.

**Supplementary Figures**

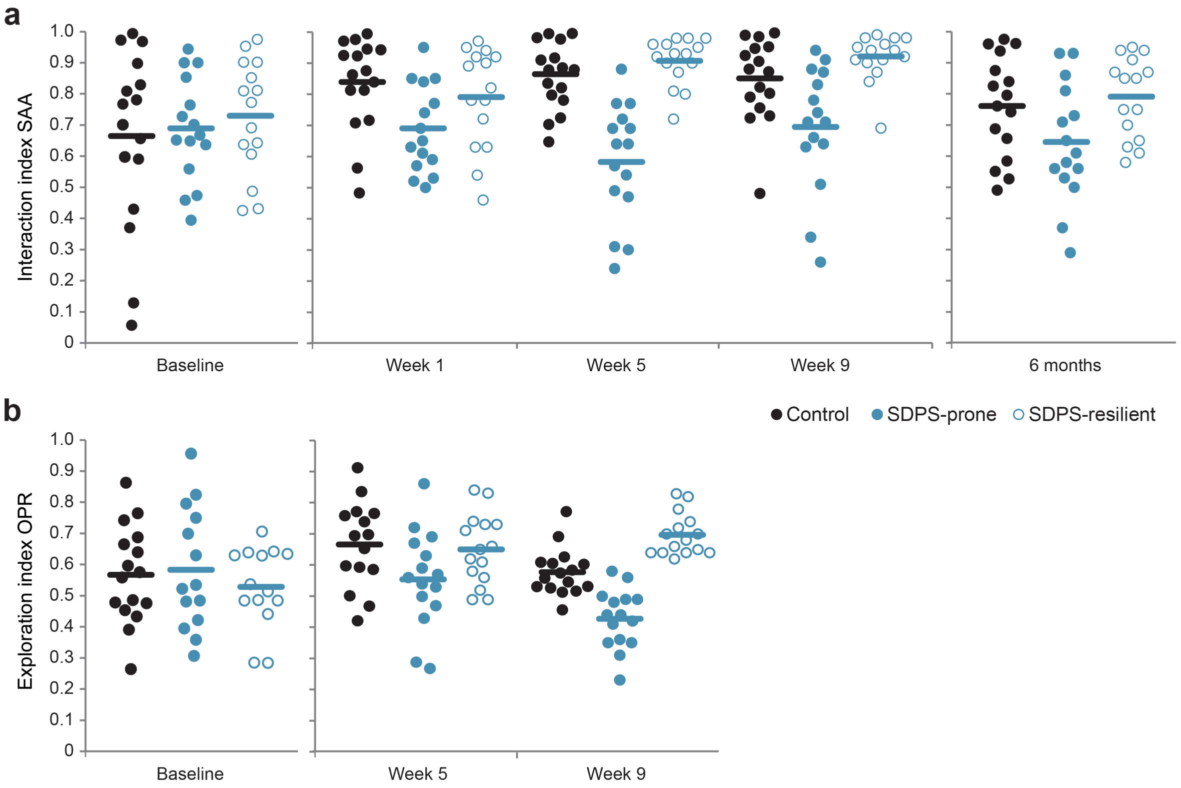


**Fig. S1. Individual scores for social behaviour and cognition over time.** (**a**,**b**) Affective and cognitive performance was assessed at different time points during the experimental schedule. Before social defeat, acutely after (w1), at one month (w5), two months (w9) and 6 months (~24w) following the last defeat exposure, rats were subjected to the Social Approach-Avoidance (SAA) task (a). Similarly, rats were subjected to the Object Place Recognition (OPR) task before social defeat, at one month (w5) and at two months (w9) following the last defeat exposure (b). Individual performance of SDPS-prone and -resilient rats and that of controls in each test, as well as group means (horizontal lines) are indicated.

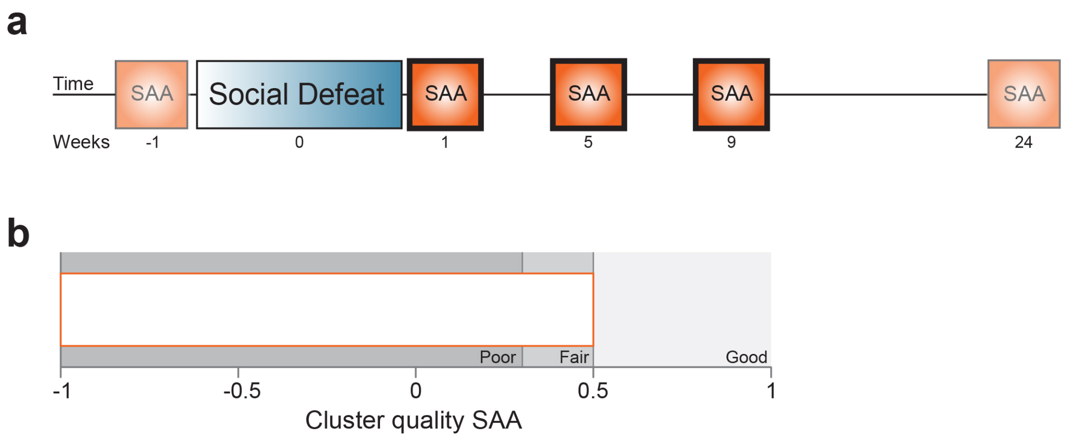


**Fig. S2**. **Cluster analysis of social behaviour: Overall model fit. a)** Experimental time-line of temporal profiling of affective function. The social approach-avoidance (SAA) task was assessed in the weeks (w-1, w1, w5, w9, w24) before/after 5 daily defeat sessions (week 0). **b)** Cluster quality after including individual data (n=48) obtained from the acute ($SAA_{w1}$) test. A decrease in overall model fitting (Fair, 0.50, orange open bar) was observed, when compared with the analysis limited to $SAA_{w5}$ and $SAA_{w9}$ tests (*cf.* Fig. 1). For comparison, poor (0.30) and fair (0.50) model fitting are depicted (grey shading).

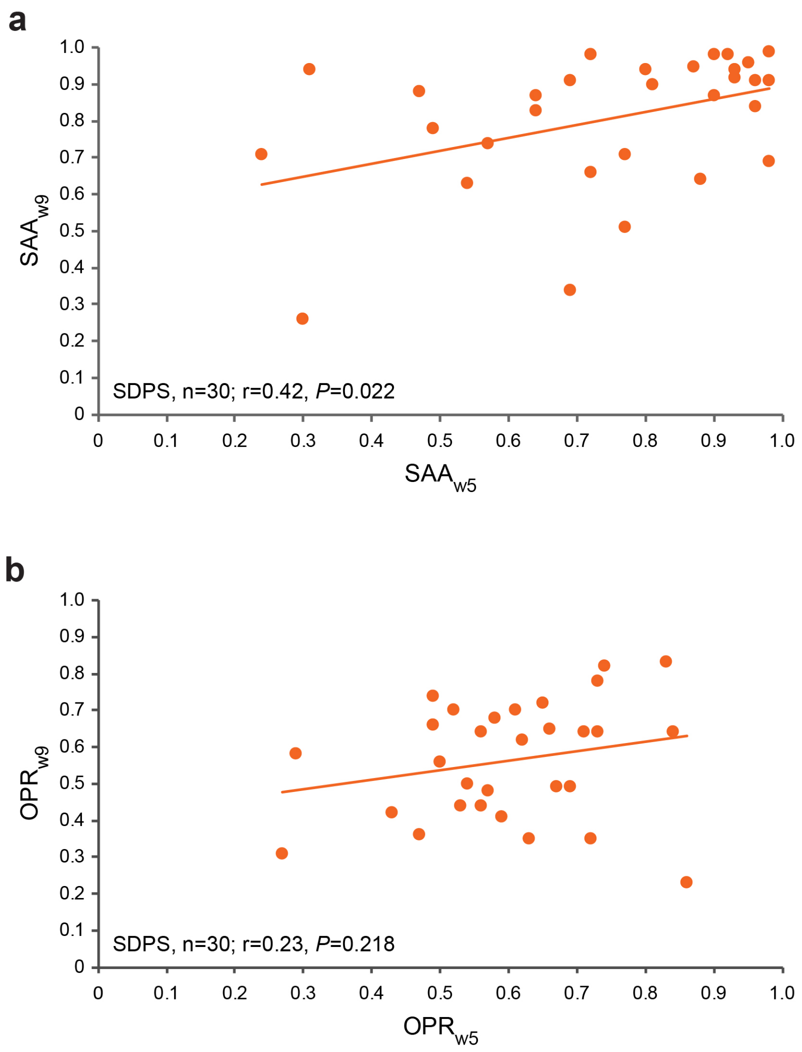


**Fig. S3. Social behaviour and cognition following SDPS: time-dependency.** Affective (social approach-avoidance -SAA) and cognitive (object place recognition -OPR) performance was assessed at weeks 5 (w5) and 9 (w9) following the last defeat session. Performance was plotted on an rat by rat basis against the two time points. **a)** A positive correlation between w5 and w9 was detected for the SAA task, indicating that social behaviour remained stable throughout the experimental period. **b)** Unlike SAA performance, no clear correlation between the two time-points examined was found for the OPR task. These results are in accordance with the temporally delayed emergence of cognitive deficits after SDPS (*cf.* Fig. 1). Spearman's correlation coefficient (r) and corresponding *P*-values are indicated. n=number of animals.

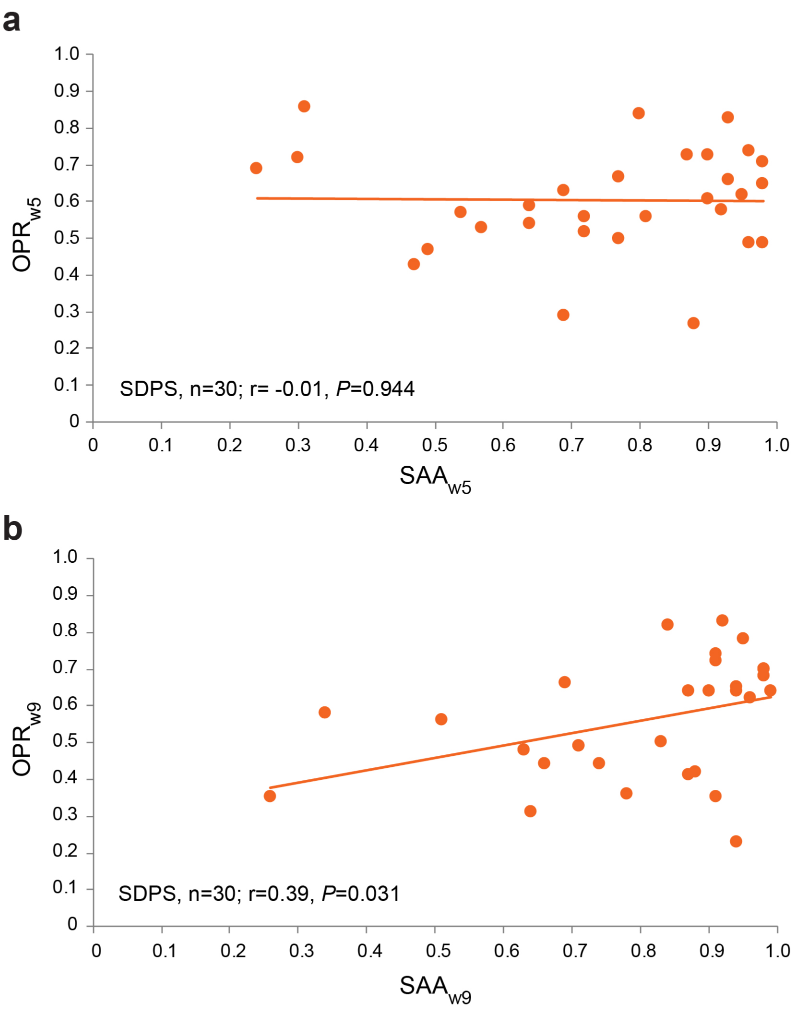


**Fig. S4. Social behaviour and cognition following SDPS: task-dependency.** Affective (social approach-avoidance; SAA) and cognitive (object place recognition; OPR) performance was assessed at weeks 5 (w5) and 9 (w9) following the last defeat session, and was plotted for the two tasks. **a)** At week 5 after defeat, no correlation between SAA and OPR performance was detected, highlighting the temporal discordance in SDPS-induced symptoms of the affective and the cognitive domain (*cf.* Fig. 1; Fig. S3). **b)** On the other hand, the positive correlation between SAA and OPR tasks verified the coincidence of impaired social behaviour and cognitive symptoms at week 9 after defeat. Spearman's correlation coefficient (r) and correspondent *P*-values are indicated. n=number of animals.

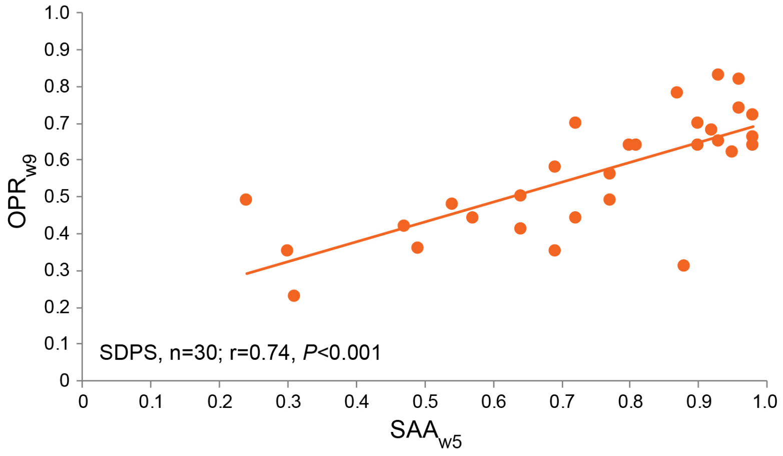


**Fig. S5. Early social avoidance predicts later spatial memory impairment.** Affective (social approach-avoidance; SAA) and cognitive (object place recognition; OPR) performance was assessed at weeks 5 (w5) and 9 (w9) following the last defeat session. Performance at $SAA_{w5}$ and $OPR_{w9}$ tests was the strongest predictor for affective and cognitive vulnerability to the effects of SDPS (*cf.* Fig. 1). Verifying this temporal development of the depressive-like symptoms in the two domains, a strong positive correlation between $SAA_{w5}$ and $OPR_{w9}$ performance was detected. These data indicate that impaired social behaviour predicts later cognitive symptoms after SDPS. Spearman's correlation coefficient (r) and correspondent *P*-value are indicated. n=number of animals.

## Reference list

1 Riga, D. *et al.* A sustained depressive state promotes a guanfacine reversible susceptibility to alcohol seeking in rats. *Neuropsychopharmacology : official publication of the American College of Neuropsychopharmacology* **39**, 1115-1124, doi:10.1038/npp.2013.311 (2014).

2 Riga, D., Theijs, J. T., De Vries, T. J., Smit, A. B. & Spijker, S. Social defeat-induced anhedonia: effects on operant sucrose-seeking behavior. *Front Behav Neurosci* **9**, 195, doi:10.3389/fnbeh.2015.00195 (2015).
